# Reciprocal Interplay Between Fibrillar Collagens and Collagen-Binding Integrins: Implications in Cancer Progression and Metastasis

**DOI:** 10.3389/fonc.2020.01488

**Published:** 2020-08-18

**Authors:** Isabelle Bourgot, Irina Primac, Thomas Louis, Agnès Noël, Erik Maquoi

**Affiliations:** Laboratory of Tumor and Development Biology, GIGA-Cancer, University of Liège, Liège, Belgium

**Keywords:** fibrillar collagens, extracellular matrix, integrins, cancer, metastasis

## Abstract

Cancers are complex ecosystems composed of malignant cells embedded in an intricate microenvironment made of different non-transformed cell types and extracellular matrix (ECM) components. The tumor microenvironment is governed by constantly evolving cell-cell and cell-ECM interactions, which are now recognized as key actors in the genesis, progression and treatment of cancer lesions. The ECM is composed of a multitude of fibrous proteins, matricellular-associated proteins, and proteoglycans. This complex structure plays critical roles in cancer progression: it functions as the scaffold for tissues organization and provides biochemical and biomechanical signals that regulate key cancer hallmarks including cell growth, survival, migration, differentiation, angiogenesis, and immune response. Cells sense the biochemical and mechanical properties of the ECM through specialized transmembrane receptors that include integrins, discoidin domain receptors, and syndecans. Advanced stages of several carcinomas are characterized by a desmoplastic reaction characterized by an extensive deposition of fibrillar collagens in the microenvironment. This compact network of fibrillar collagens promotes cancer progression and metastasis, and is associated with low survival rates for cancer patients. In this review, we highlight how fibrillar collagens and their corresponding integrin receptors are modulated during cancer progression. We describe how the deposition and alignment of collagen fibers influence the tumor microenvironment and how fibrillar collagen-binding integrins expressed by cancer and stromal cells critically contribute in cancer hallmarks.

## Introduction

Cancer progression is a highly dynamic process implicating distinct features responsible for tumor growth and metastatic dissemination. These features comprise sustained proliferative signals, evading growth suppression, resisting cell death, stimulating angiogenesis, activating invasion and metastasis, deregulating cell metabolism and avoiding immune destruction (1). These hallmarks are obtained through reciprocal interactions between cellular and non-cellular components of tumors, which define the tumor microenvironment (TME). The critical role of the TME in cancer progression has been initially recognized in 1863 by Virchow, who first described that malignancies occurred at sites of chronic inflammation (2). In 1889, the “seed and soil” hypothesis was proposed by Paget suggesting that the TME was important for tumor progression (3). Solid tumors are very heterogeneous and resemble a complicated organ whose complexity approaches and may even exceed that of normal healthy tissues (4). These tumors contain a complex mixture of non-cancerous cellular elements including blood and lymphatic vessels, pericytes, cancer-associated fibroblasts (CAFs), mesenchymal cells, immune/inflammatory cells and nervous network. Non-soluble or semi-soluble substances, such as the extracellular matrix (ECM), and soluble substances, such as interstitial fluids, cytokines, chemokines, growth factors, and metabolites constitute the acellular components of the TME (5–7). Physico-chemical parameters including interstitial pressures, oxygen level, and pH/redox potential represent additional critical characteristics of the TME. It is important to note that the TME is relatively abundant in comparison to cancer cells, with a proportional ratio nearly always in favor of the TME. In solid tumors, including breast and pancreatic tumors, the TME constitute up to 90% of the tumor mass (8–10). The TME is also characterized by a compositional and spatial heterogeneity, which varies greatly across tumor types, amongst patients with a given cancer type, and across distinct lesions in a given patient.

The ECM, which provides architectural support and anchorage for the cells, is composed of a complex meshwork of highly cross-linked components, including fibrous proteins, glycoproteins, proteoglycans, and polysaccharides (11–13). The biomechanical and biochemical properties of the ECM regulate cell survival, proliferation, differentiation and motility by ligating specific cell surface receptors including integrins, discoidin receptors and syndecans (14, 15). Besides these signaling properties, the ECM also play essential roles in tissue function by providing a structural and mechanical support for tissue integrity. It also influences the availability of cytokines and growth factors and maintains the hydration of the microenvironment. The structure of the ECM is highly dynamic and continuously remodeled through ECM deposition, degradation, or modification (16). Collagens are the most abundant components of the ECM, however, their structure and composition differ across various tissue types (17, 18). For example, the basement membrane, a well-structured matrix underlining epithelial and endothelial cells and separating them from the interstitial stroma, mainly consists of collagens type IV and VIII, while the interstitial stroma is mostly composed of fibrillar collagens type I, II, and III (19, 20).

**Figure 1:**
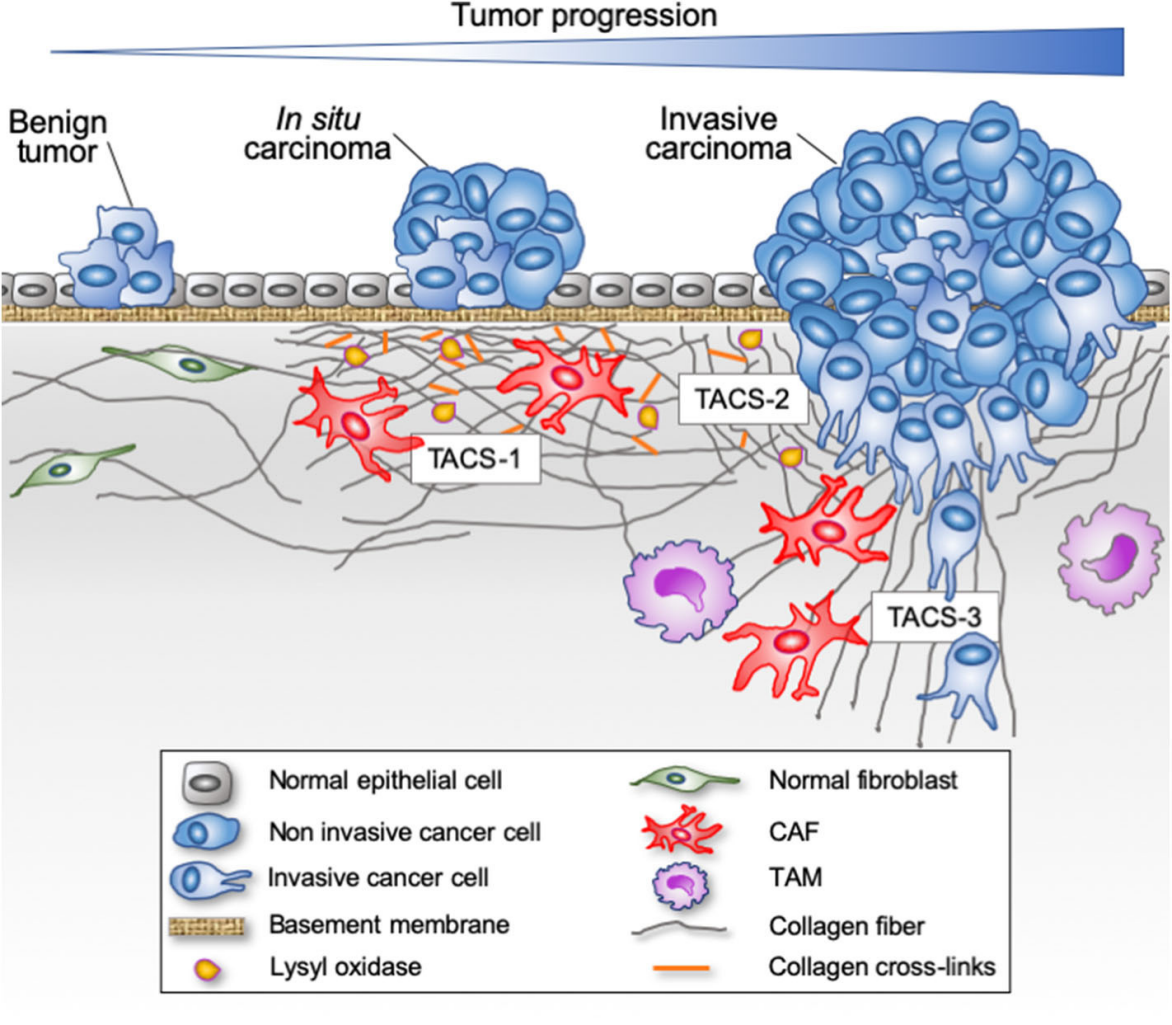
Evolution of fibrillar collagen organization during tumor progression. The transition from a benign tumor to an *in situ* carcinoma is associated with a progressive reorganization of the tumor microenvironment. Epithelial cells are separated from the stroma by a continuous basement membrane. Tumor-derived paracrine signals promote a desmoplasic reaction characterized by the activation of the resident fibroblasts into cancer-associated fibroblasts (CAFs) able to secrete and reorganize the collagen fibers (cross-linking), thereby increasing the stiffness of the stroma. Tumor-associated macrophages (TAMs) are also recruited and contribute to collagen remodeling. When invasive cancer cells have breached the basement membrane, they become confronted with the collagen-rich desmoplasic stroma. The collagen fibers located in the vicinity of the invading cancer cells can be organized parallel to the tumor border (Tumor Associated Collagen Signature—TACS-2) or linearized and oriented perpendicular to the tumor border (TACS-3), thereby promoting the migration of invading cancer cells.

Abnormal deposition and crosslinking of fibrillar collagens has serious repercussion on tissue homeostasis. Solid tumor's ECM is typically more rigid than normal tissue as a result of the overexpression of several ECM components, including collagens I, II, III, V, IX, XI, and heparan sulfate proteoglycans as well as ECM-crosslinking enzymes such as lysyl oxidases (21, 22). This accumulation generates a stroma characterized by a dense meshwork of fibrillar proteins (Figure 1), which progressively causes tissue stiffening, a hallmark of many cancers, such as breast, pancreatic and prostate cancers (23, 24). Stiffened ECM increases integrin-mediated mechanotransduction related signals, thereby promoting cancer cell survival, proliferation, and invasion (25–27). In epithelial cancers, the transition from an *in situ* to an invasive carcinoma with associated high mortality is characterized by the focal degradation of the basement membrane (28). The breaching of the basement membrane by malignant cells is significantly influenced by the stiffness of the associated interstitial ECM (29). Tumor cell invasion through the basement membrane exposes malignant cells to a completely different microenvironment mostly dominated by the fibrillar collagens of the interstitial stroma (Figure 1). This new microenvironment rewires tumor cells by altering gene expression, cell proliferation, apoptosis, migration and survival, thereby directly affecting the hallmarks of cancer (30–33).

In this review, we describe the different fibrillar collagens and highlight how these proteins and their corresponding integrin receptors are modulated during cancer progression. We describe how the deposition and alignment of collagen fibers influence the TME and how integrin binding fibrillar collagen expressed by cancer and stromal cells represent critical players in cancer hallmarks. A brief overview of the different imaging techniques used to visualize and analyze fibrillar collagens is also provided.

## Collagens

Collagens, which constitutes up to 30% of the total protein mass in the human body, represent the most abundant proteins in mammals and are characteristic of the metazoan family (34, 35). In the human genome, 44 collagen genes code for polypeptidic chains and are combined in diverse ways to form 28 collagen types, numbered with roman numerals in vertebrates (I–XXVIII) (36, 37).

**Figure 2:**
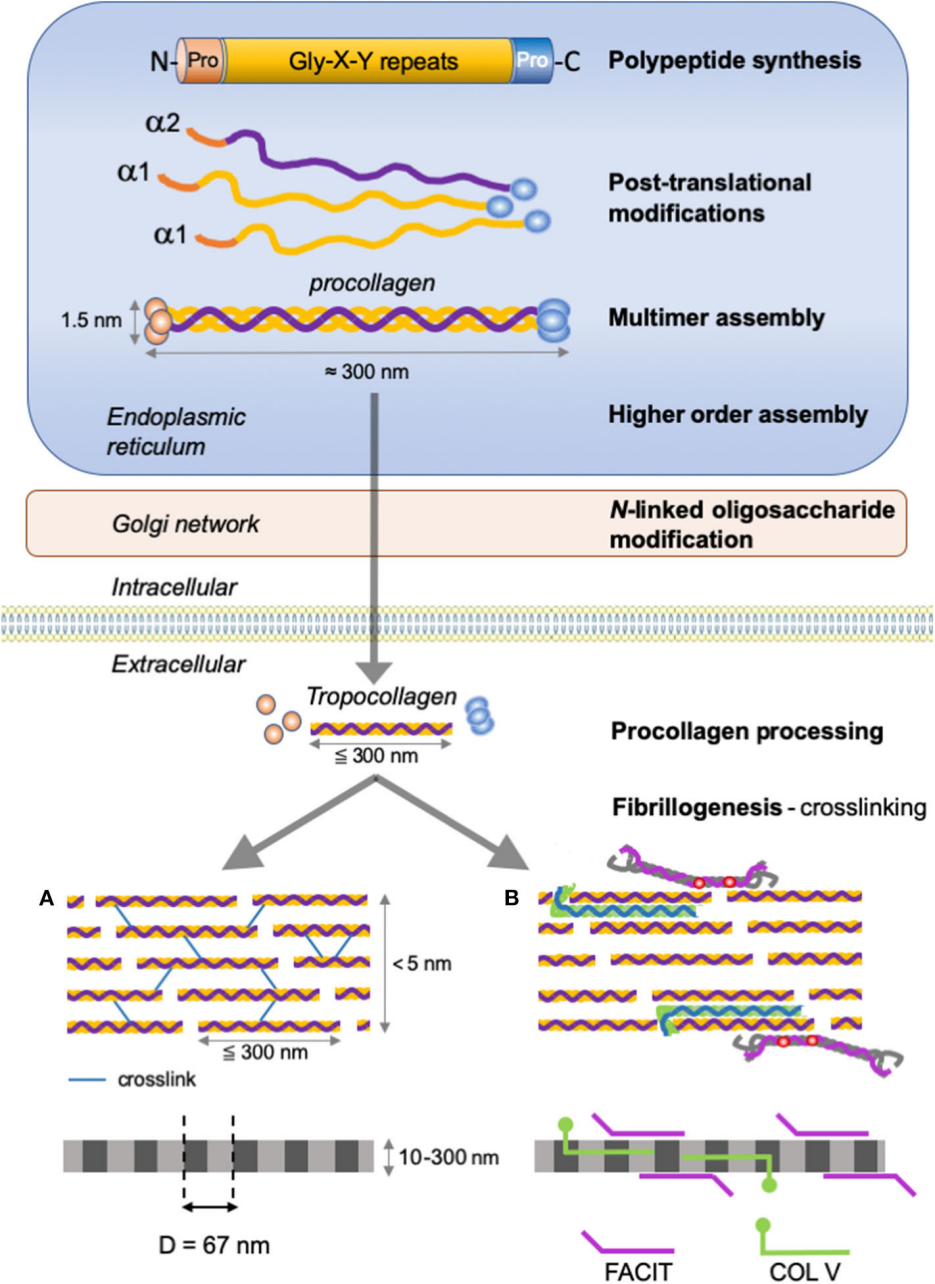
Type I collagen supramolecular assembly pathway. The standard fibrillar collagen molecule is characterized by N- and C-terminal propeptide sequences, which flank a series of Gly-X-Y repeats (where X and Y represent any amino acids but are frequently proline and hydroxyproline). These form the central triple helical structure of procollagen and collagen. Three precursor α-chains (two α1 and one α2) are co-translationally translocated into the endoplasmic reticulum lumen, where specific post-translational modifications occur. Three collagen α-chains associate specifically via their C-terminal domains to form heterotrimers. The helical collagens are trafficked via the Golgi network to the plasma membrane, and secreted into the extracellular space as precursor forms, called procollagens, with N- and C-terminal non-collagenous domains. These domains are removed by the action of specific proteases, and the collagens are assembled into dense fibrils with a characteristic D-periodicity of about 67 nm **(A)**. The fibril is stabilized by covalent lysine- and hydroxylysine-derived crosslinks. In addition to fibrillar collagen, other collagens, such as type V and FACIT collagens, are incorporated into the fibril structure **(B)**. Type V collagen is inserted between strands of the microfibril, and FACIT collagens cling to the surface of the microfibril and work to stabilize higher order structures. Adapted from (38).

The term “collagen” is commonly used to refer to homotrimeric and heterotrimeric proteins formed by three polypeptide chains (α-chains). A characteristic feature of all collagens is the presence of a tight right-handed triple helix composed of three polypeptides α-chains forming a functional collagen molecule (Figure 2) (36, 39, 40). The triple helix motif can represent up to 96% of the collagen structure (for collagen I) to <10% (collagen XII) (41). Collagen molecules are made up of a tight right-handed helix composed of three α-chains, each of which contains one or more regions characterized by the repeating amino acid motif (Gly-X-Y)_n_, with proline and 4-hydroxyproline amino acids often found at the X and Y positions, respectively (42). The presence of a glycine residue in every third position is required for the assembly into a triple helix. Indeed, the tight packing of the three α-chains near the common axis induces steric constraints on every third amino acid position and only glycine, the smallest amino acid can accommodate without any chain deformation. Consequently, the glycine residues are positioned in the center of the triple helix, where they stabilize the structure (42–44). Some collagen molecules assemble as homotrimers, whereas others assemble as heterotrimers composed of two or three distinct α-chain types. For example, type I collagen contains two identical α1 chains and one α2 chain, [α1(I)]_2_ α2(I). Each α-chain forms an extended left-handed helix with a pitch of 18 amino acid per turn (45).

**Figure 3:**
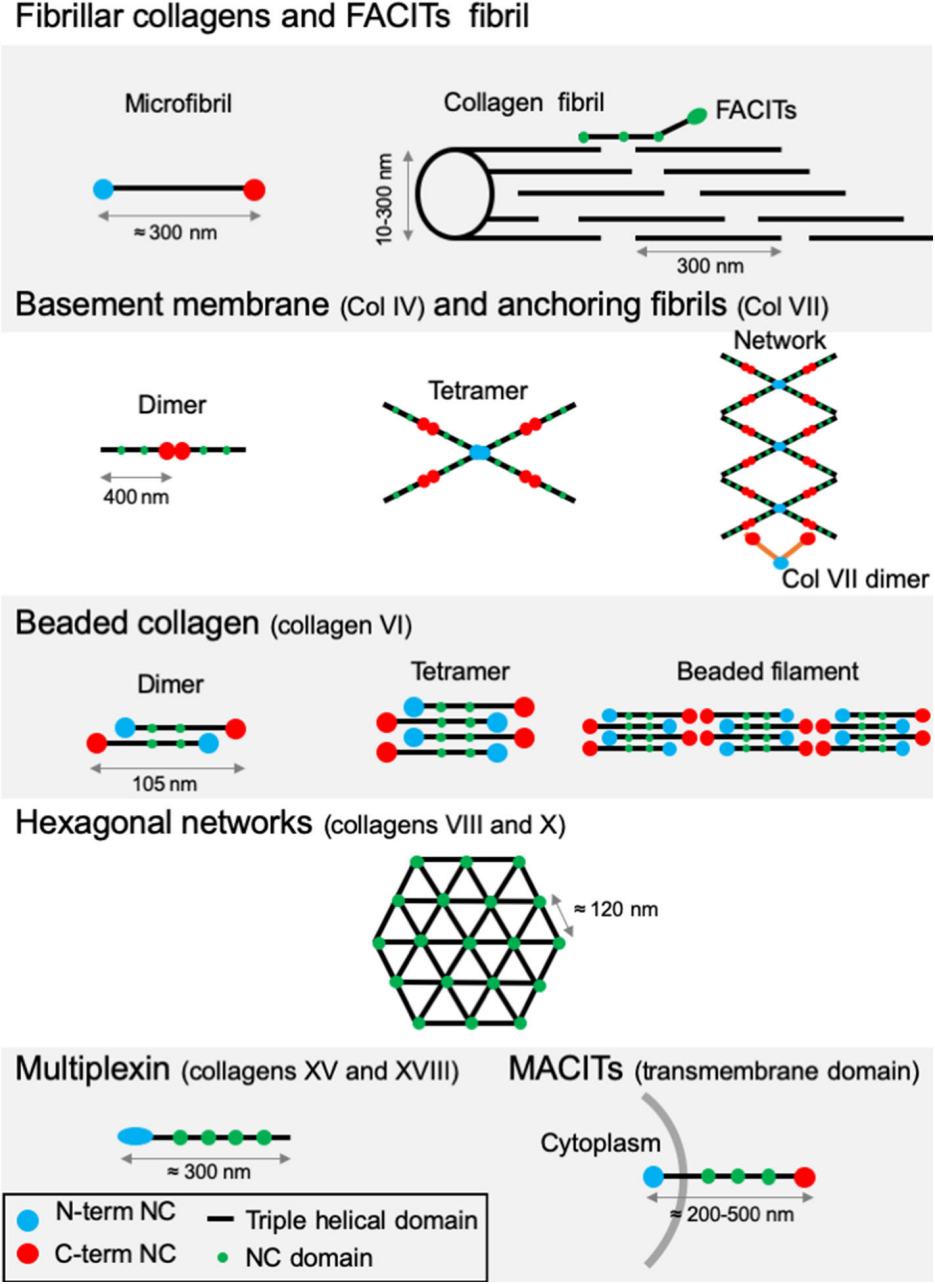
Supramolecular structures formed by some archetypal collagens. *Fibrillar collagens and FACITs fibrils*: the association of mature protomers together leads to the formation of microfibrils which in turn assemble into fibrils. FACITs protomers attach at the surface of fibers with the C-terminal part protruding and regulate fibrillogenesis. *Basement membrane and anchoring fibrils*: formation of type IV collagen dimer occurs by the association of two protomers through their globular NC C-terminal domain. Dimers interact together through their N-terminal domains to constitute tetramers. Networks are the result of the two first steps linked to additional lateral interactions between the molecules. Dimers of type VII collagen interact with the network of type IV collagen. *Beaded collagen*: an association between the type VI collagen dimer and tetramer takes place inside the cells. Connection of tetramers leads to the formation of long filaments called “beaded filaments” according to their appearance in electron microscopy. *Hexagonal networks*: collagens VIII and X form hexagonal networks in Descemet's membrane and in hypertrophic cartilage, respectively. *Multiplexin*: collagens XVIII and XV are found in basement membrane. *MACITs*: transmembrane collagens (XIII, XVII, XXIII, and XXV). The N-terminal NC domain (N-terminal NC) is located inside the cell, whereas the triple helix region is extracellular. NC, non-collagenous domain. Adapted from (41).

All members of the collagen family present diverse supramolecular assemblies in the ECM and the capacity to bind to cell surface receptors or other protein glycosaminoglycans (GAGs) (41). Their size, function and tissue distribution may vary considerably from networks to fibrils. The existence of several α-chains, different supramolecular structures for each collagen type, diverse molecular isoforms as well as the use of alternative promoters and alternative splicing highlight the complexity and diversity of the collagen family (46, 47). Based on this variability, vertebrate collagens have been classified into different families. The most abundant collagen family, with about 90% of the total collagens, is represented by the fibrillar collagens (Table 1). In human, the main fibrillar collagens include types I, II and III (major fibrillar collagens), V and XI (minor fibrillar collagens), and the more recently discovered types XXIV and XXVII (49, 50). Fibrillar collagens are characterized by the presence of uninterrupted triple-helical domains of about 300 nm, forming large extracellular fibrils. Type I collagen represents the archetypal fibrillar collagen due to the presence of a trimeric structure and the absence of imperfection in the triple helix. This molecular organization contrasts with that of other collagen families, which present interruptions in the triple helix or do not assemble into fibrils (Figure 3). Network-forming collagens and anchoring fibril (ex: type IV and type VII collagens) have extended triple helices (>350 nm) with imperfections in the Gly-X-Y repeat sequences. Types VI, VIII, and X collagen are characterized by the presence of short continuous triple-helical domains. Type VI belongs to beaded filament (51), while types VIII and X form hexagonal networks (40). FACITs (Fibril-Associated Collagens with Interrupted Triple helices) are referred to as fibril-associated molecular bridges. They include collagen IX, XII, XIV, XVI, XIX, XX, XXI, XXII, and XXVI, which are associated to the surface of different collagen fibrils (Figures 2, 3) (40, 52). Collagens type XIII, XVII, XXIII, and XXV represent the MACIT, Membrane-Associated Collagens with Interrupted Triple helices, and function as cell surface molecules with a transmembrane domain. Finally, the multiplexin family (collagens XV and XVIII) is characterized by the presence of multiple triple helix domains interrupted by non-collagenous domains (36).

**Table 1:** Molecular chain compositions of human fibrillar and FACIT collagens.

**Classes**	**Type**	**Molecular composition**	**Remarks**
Fibrillar collagen	I	[1α(I)]_2_α2(I)	Fibrils composed of uninterrupted triple helical domains that are primarily found in skin, bone, tendons and ligaments, cartilage, cornea
	II	[α1(II)]_3_	
	III	[α1(III)]_3_	
	V	[α1(V)]_2_, α2(V); [α1(V)]_3_; α1(XI)α2(V)α3(XI)	
	XI	α1(XI)α2(XI)α3(XI)	
	XXIV	[α1(XXIV)]_3_	
	XXVII	[α1(XXVII)]_3_	
FACIT collagen	IX	[α1(IX), α2(IX), α3(IX)]	Fibril-associated collagens with interrupted triple helices: forming molecular bridges to the surface of collagen fibrils (e.g., IX, XII, XIV, XVI, XIX, XX, XXI)
	XII	[α1(XII)]_3_	
	XIV	[α1(XIV)]_3_	
	XVI	[α1(XVI)]_3_	
	XIX	[α1(XIX)]_3_	
	XX	[α1(XX)]_3_	
	XXI	[α1(XXI)]_3_	
	XXII	[α1(XXII)]_3_	

*Adapted from (41, 48)*.

In this review, we will focus on the fibrillar and fibrillar-associated collagens. For further information on non-fibrillar collagens, the reader is referred to other publications (18, 41, 53, 54).

## Fibrillar Collagens

A key characteristic of fibrillar collagens is their ability to assemble and to form highly orientated supramolecular aggregates. Type I collagen represents 90% of the total collagen. It constitutes the major collagen of the skin, tendons, ligaments, cornea, and other interstitial connective tissues. Type I collagen is mostly incorporated into a composite containing either type III collagen in skin and reticular fibers (55) or type V collagen in bone (56). The biomechanical properties of these composites (e.g., torsional stability, tensile strength, torsional stiffness) ensure the stability and integrity of these tissues (57, 58). The production of fibrillar collagens requires several intracellular and extracellular post-translational steps, which lead to the formation of elongated, cable-like striated fibril structures that are capable of withstanding tensile forces. This illustrates the close relationship between the three-dimensional protein structure and the role of the resultant ECM.

## Biosynthesis and Arrangement in Superstructure

Collagen biosynthesis is a complex multistep process starting with the synthesis of long α-chains precursors called *procollagens* (Figure 2). The growing peptide chains are co-translationally transported into the rough endoplasmic reticulum where multiple co- and post-translational modifications take place prior to the formation of the triple-helical procollagen.

The fibrillar collagen precursor is synthetized as a multidomain precursor constituted by a long triple helical COL1 domain (about 300 nm in length) flanked by two non-collagenous domains: a specific trimeric C-terminal NC1 domain (C-telopeptide and a C-propeptide) and a N-terminal NC2 domain (a short, non-helical, N-telopeptide and an N-terminal propeptide) (Figure 2). These non-collagenous domains represent important structural components: the C-propeptide plays an essential function in the initiation of triple helix formation and the N-propeptide is implicated in the regulation of primary fibril diameters (38).

Extensive post-translational modifications, including hydroxylation of proline and lysine residues, glycosylation of lysine and hydroxylysine residues, occur prior to the formation of the triple helix (36). These modifications are stopped by the formation of the triple helix. Hydroxylation of proline and lysine residues are catalyzed by prolyl 3-hydroxylase, collagen prolyl 4-hydroxylase (C-P4Hs), and lysyl hydroxylase, respectively. These proline and hydroxyproline amino acids can comprise up to 20% of the molecule. Prolyl hydroxylation (in Y position) is essential for intramolecular hydrogen bonding and to increase the thermal stability of the triple helix. In fibril forming collagens ~50% of prolines are 4-hydroxylated; the extent of these hydroxylations varies between tissues and collagen types (59). Finally, glucosyl and galactosyl residues are added to the hydroxyl groups of hydroxylysine residues by the hydroxylysyl galactosyltransferase and galactosylhydroxylysyl-glucosyltransferase, respectively. Thus, the three α-chains are maintained together by intramolecular hydrogen bonds (high proline and 4-hydroxyproline content-dependent) and electrostatic interactions involving lysine and aspartate (42, 60–62). Highly ordered hydration networks surround the triple helices allowing a close packaging along the central axis of the molecule. Following hydroxylation and glycosylation processes, C-propeptide domains have an essential function (as nucleus) in the initiation and folding of the triple helix (43, 44, 63, 64). These domains allow the proper selection and alignment of collagen α-chains via intrachain disulfide bonds between the C-terminal propeptides of three procollagens sequences. The central portions of the chains zipper from C- to N-terminus to form the triple helix (65).

Many chaperone proteins including the heat shock protein 47 (HSP47) may influence and guide the formation of the triple helix during procollagen assembly (66). The binding of HSP47 enables the effective assembly and folding of the procollagen chains and facilitates the stabilization of procollagen triple helix at body temperature.

Once assembled in the endoplasmic reticulum, procollagen molecules are packaged in the Golgi for export into the ECM. Once released in the extracellular space, the procollagen is cleaved to form the mature triple-helical collagen or tropocollagen. Cleavage of N- and C-propeptides allows spontaneous fibrillogenesis in the extracellular environment. During or following exocytosis, extracellular proteinases, the procollagen N-proteinases (identified as the ADAMTS-2, -3, -14) and procollagen C-proteinases (identified as the BMP-1/tolloid proteinases) remove the N- and C-terminal propeptides (67–74). The removal of these propeptides exposes the telopeptides (short non-triple helical extensions of the polypeptide chains), which become binding sites for further covalent crosslinking during fibrillogenesis (75). The complete removal of the propeptides from collagen I, II and III fibrils allows fibrillogenesis in the extracellular space (76). In some cases, propeptides terminal-ends remain attached or partially attached, thereby affecting the solubility of the procollagen in the extracellular space, inhibiting premature fibril assembly or influencing fibril shape and diameter. For example, the uncleaved N-propeptides of collagen type V and XI influence fibril growth by sterically limiting lateral molecule addition (77).

After processing, the mature protein (*tropocollagen)*, consists almost entirely of a triple-stranded helix and is considered as the building block for higher order fibrils and fibers. The self-assembly of tropocollagen monomers (300 nm long, 1.4 nm diameter) results in the formation of collagen microfibrils with a quarter-stagger axial D periodicity of 67 nm long repeats to create the characteristic striation observed in collagen-containing tissues (69, 78). The staggered arrangement optimizes electrostatic and hydrophobic interactions and allows the formation of covalent intermolecular cross-links between lysine/hydroxylysine residues of helical and neighboring non-helical regions thereby stabilizing the collagen fibrils (75, 79, 80). This process is initiated by the oxidative deamination of lysyl and hydroxylsyl residues in the N- and C-terminal telopeptide regions catalyzed by enzymes of the lysyl oxidase family (LOX) (81). The newly generated aldehyde group forms crosslinks with the (hydroxy)lysines in the triple-helical region of neighboring molecules. These links are crucial to confer the mechanical characteristics of collagen-containing tissues (82–84).

The short primary fibrils are unilaterally elongated via a multistage process including nucleation and organization to form intermediate-sized microfibrils. Once assembled, collagen microfibrils grow into fibrils through longitudinal and axial increase (77, 85). The assembly can also be regulated by many collagen binding proteins such as the FACITs (Figure 2), which integrate into the fibrils, selectively altering the surface properties of the collagen fibril as well as the interactions with accessory ECM molecules including the small leucine-rich repeat proteoglycans (78, 86).

## Implications of Fibrillar Collagens in Cancer Progression

In 1986, Dvorak reported an association between the TME and wound healing, suggesting that tumors behave as wounds that do not heal (87). In agreement with this concept, a fibro-inflammatory microenvironment has been shown to play critical roles in supporting tumor progression (88). Indeed, compelling studies suggest that a normal microenvironment prevents premalignant cells from progressing into cancer, whereas an abnormal or wound repair–associated microenvironment can be tumor-promoting (88). Reacting to a disruption in tissue homeostasis, resident fibroblasts are progressively activated into CAFs, which represent the most frequent cell type in the TME of many carcinomas, including pancreas, breast, and hepatic carcinomas (89, 90). During cancer progression, fibroblast activation and expansion is induced (Figure 1), such that low-grade premalignant lesions are already surrounded by areas of fibrosis (91). As these CAFs coevolve with the advancing cancer, they take on diverse functions that support tumor progression (92). In that context, CAFs represent key players in tumor fibrosis (also called desmoplasia), which is defined as a fibrotic state characterized by an excessive synthesis, deposition and remodeling of fibrillar collagens in the surroundings of the tumor (22, 93). Increasing evidence suggests heterogeneity among CAFs. Single-cell RNA-seq analyses of different mouse and human tumors highlighted the existence of at least three CAF populations (92, 94, 95). Among these, a population of CAFs has been coined myofibroblastic CAFs or matrix CAFs (mCAFs) and abundantly produces a wide variety of ECM components including type I and type III fibrillar collagens, matrix-modifying enzymes such as LOX as well as a contractile phenotype. Consistent with its role as a key regulator of fibrogenic gene expression and the myofibroblast state, TGF-β signaling promotes the mCAF phenotype (96, 97).

By exerting contraction forces on collagen fibers, CAFs induce the reorientation and alignment of fibrillar proteins. The dense collagen network is also crosslinked, thereby increasing matrix stiffness.

Atomic force microscopy analysis of a murine model of spontaneous breast cancer (MMTV-PyMT mice) revealed that while the elastic modulus of the normal mammary gland was about 400 Pa, it increased to 1,200 and 3,000 Pa in pre-malignant and malignant tumors, respectively (98). In human breast, the stiffness of the normal and non-invasive stroma is around 400 Pa. In sharp contrast, the invasive regions of aggressive tumors were much stiffer (>5 kPa). Interestingly, the tumor invasion front appears stiffer than the tumor core tissue (99).

This desmoplasia-associated collagen remodeling elicits biochemical and biophysical cues which influence stromal and tumor cell properties, providing crucial physical guidance facilitating cell migration, invasion, and metastasis through reciprocal interactions between the cells and the ECM (21, 26, 100–105).

Several key biophysical parameters of type I collagen matrix play a role in cancer cell migration, in particular the mechanical properties, the pore size, the density and orientation of cell adhesion sites presented by the collagen fibers, and the local direction of the fibers (106).

Collagen fiber diameter and pore size play key roles in cell force generation and migration (107, 108), with larger fiber diameter and pore size promoting cell force generation and migration (21).

During migration, cancer cells follow the path of least resistance, they recognize and use open pores within the matrix. A correlation between cell-migration speed and pore size has been shown (107, 109). Cancer-cell migration is also positively correlated with the stiffness of the tumor and associated stromal matrix (110, 111). However, fiber alignment appears as the best predictor of cell speed in collagen matrix.

The mode of cell migration is also dictated by the microstructure of the matrix. At low collagen densities, when the matrix pore size is larger than the nucleus of the migrating cell, matrix cleavage is not critical and cells move rapidly using pseudopodial protrusions in an amoeboid mode of migration (107, 112). Increased deposition of fibrillar collagens may lead to decreased porosity near the tumor. At high collagen densities, when the pore size is significantly smaller than the nucleus of the cell, cell movements rely more extensively on the cleavage of collagen fibers by secreted and cell membrane-associated collagenolytic enzymes including matrix metalloproteinases (MMPs), cathepsins, and serine proteinases. In this case, tumor cells undergo a more mesenchymal mode of migration: they use proteinase-assisted invadopodia to open the pore to the necessary size and move through them by transmitting forces via adhesion. However, some cells are able to migrate through small pores in the absence of matrix remodeling by disrupting and subsequently repairing their nuclei (113).

The proteinase-assisted migration generates channel-like tracks (3–30 μm in diameter and 100–600 μm in length) in the matrix (114). These migration tracks opened by path-finding cells can subsequently be used by several following cancer cells (109, 115).

CAFs also play an active role in promoting cancer cell infiltration into the tumor stroma by taking the lead and forming tracks in which tumor cells follow (116). The remodeling of fibrillar collagens by CAFs and/or cancer cells may lead to heterogeneities in density and network organization.

The alignment of the fibrillar components of the ECM has a strong influence on the direction and speed of migrating cells (16, 117). Experimental models have demonstrated that cancer cells invade more efficiently through *in vitro* engineered lattices of linear type I collagen than through disorganized lattices (105, 106, 118). *In vivo*, local tumor cell invasion was oriented along radially aligned collagen fibers. These reorganized collagen fibers are called tumor-associated collagen signatures (TACS, Figure 1). Three TACS corresponding to different levels of collagen fibers reorganization have been described and represent novel markers to locate and characterize tumors (103, 119–121). In TACS-1 stage, a localized increase in the deposition of collagen without obvious alignment is observed near the tumor. In TACS-2 stage (pre-invasive tissue), the collagen fibers are aligned in parallel to the tumor border. In TACS-3 stage (metastatic stage), the collagen fibers are bundled and aligned perpendicular to the tumor border. Collagen linearization is therefore considered as a key feature of metastatic carcinomas and predictive of poor prognosis in breast carcinoma and *in situ* breast ductal carcinoma (121–123). The aligned collagen fibers provide tracks not only for cancer cells but also for macrophages, thereby promoting their migration into the TME (124). Recently, WISP1, a matricellular protein secreted by cancer cells, was shown to induce collagen linearization by binding directly type I collagen, thereby promoting its linearization, independently of cell-derived mechanical tensions (125). Moreover, fibroblast activation protein (FAP), another important ECM-associated proteinase, promotes the formation *in vitro* of desmoplastic-like aligned matrices (126) and its overexpression has been associated with a poor patient outcome.

The accumulation of cross-linked collagens and the subsequent stiffening of the matrix also lead to elevated interstitial fluid pressure in the desmoplastic TME inducing resistance to treatment by decreasing chemotherapy and immunotherapy drug delivery (127).

In addition to their biomechanical contribution to cancer progression, CAF-derived collagen-rich ECMs also indirectly fuel cancer cells with amino acids. Indeed, collagen uptake and catabolism, with subsequent proline catabolism, has been shown to support cancer cell proliferation (128).

Even if CAFs are usually considered as the key ECM remodelers in the TME, both epithelial and immune cells also contribute to the synthesis and secretion of fibrillar ECM components (129). Indeed, epithelial cells are able to secrete ECM components (including type I collagen) (130–133). In colorectal cancer, tumor associated macrophages (TAMs) could even play a more important role in collagen deposition, cross-linking, and linearization than CAFs (134).

The desmoplastic reaction has been frequently associated with a poor survival of cancer patients (135–140). According to clinical data, tumor collagen content, alignment and distribution have been considered as prognostic factors related to cancer differentiation, invasion and clinical outcome in different cancers (141–143). However, the implications of fibrillar collagens during cancer progression are not restricted to the primary tumors. Despite the successful treatment of a primary tumor, dormant disseminated tumor cells may be reactivated to form actively proliferating metastatic lesions (144, 145). This reactivation is associated with the induction of fibrosis characterized by the deposition of fibrillar collagens in the metastatic microenvironment (146).

Beside the above described functions in desmoplasia, fibrillar collagens are extremely important in major steps of cancer progression. Specific chains of these collagens and procollagens act as effectors and allow modulation of key processes in cancer progression (e.g., proliferation, apoptosis, angiogenesis, invasion, and metastasis). Some studies reveal that these effects can be either pro- or anti-tumorigenic and are collagen type-dependent (147, 148). A non-exhaustive overview of the contribution of each fibrillar collagens in cancer progression is presented in Table 2.

**Table 2:** Influence of fibrillar collagen expression on cancer properties.

**Type**	**Subtype**	**Cancers**	**Implications**	**References**
COL I	COL1A1	Hepatocellular	Clonogenicity, motility, invasiveness and stemness	(149)
		Bladder	Proliferation, migration, and invasion	(150)
		Malignant astrocytoma	Invasion	(151)
		Breast	Migration	(152)
		Colorectal	Migration	(153)
		Oral squamous cell carcinoma	Proliferation and migration	(154)
		Gastric	Proliferation, migration, and invasion	(155)
COL II	COL2A1	Ovarian	Prognostic value	(156)
		Gastric	Prognostic value	(157)
			Biomarker	(158)
COL III	COL3A1	Ovarian	Lymphovascular metastasis	(159)
			Patient survival	(160)
			Drug resistance	(161)
		Bladder	Patient survival	(162)
		Glioblastoma	Patient survival	(163)
		Colorectal	Patient survival	(164)
			Proliferation	(164)
COL V	COL5A1	Ovarian	Lymphovascular metastasis	(159)
		Breast	Viability and migration	(165)
			Patient survival	(165)
		Lung	Proliferation, apoptosis, invasion	(166)
			Patient survival	(166)
		Renal	Proliferation, apoptosis, migration, invasion	(167)
			Patient survival	(167)
		Tongue squamous cell carcinoma	Patient survival and metastasis	(168)
		Breast	Prognostic value	(169)
	COL5A2	Ovarian	Lymphovascular metastasis	(159)
		Tongue squamous cell carcinoma	Patient survival	(168)
		Bladder	Patient survival	(170)
	COL5A3	Breast	Proliferation	(171)
COL XI	COL11A1	Ovarian	Patient survival	(172)
			Invasion	(172)
			Drug resistance	(173)
		Non-small cell lung carcinoma	Proliferation, migration, invasion, drug resistance	(174)
		Pan-cancer	Marker of activated CAF	(175)
		Gastric	Proliferation, migration, invasion	(176)
		Esophageal squamous cell carcinoma	Prognostic value	(177)
		Pancreas	Prognostic value	(178)
		Breast	Prognostic value	(179)
COL XXIV	COL24A1	Head and neck	Prognostic value	(180)
		Hepatocellular carcinoma	Prognostic value	(181)

**Figure 4:**
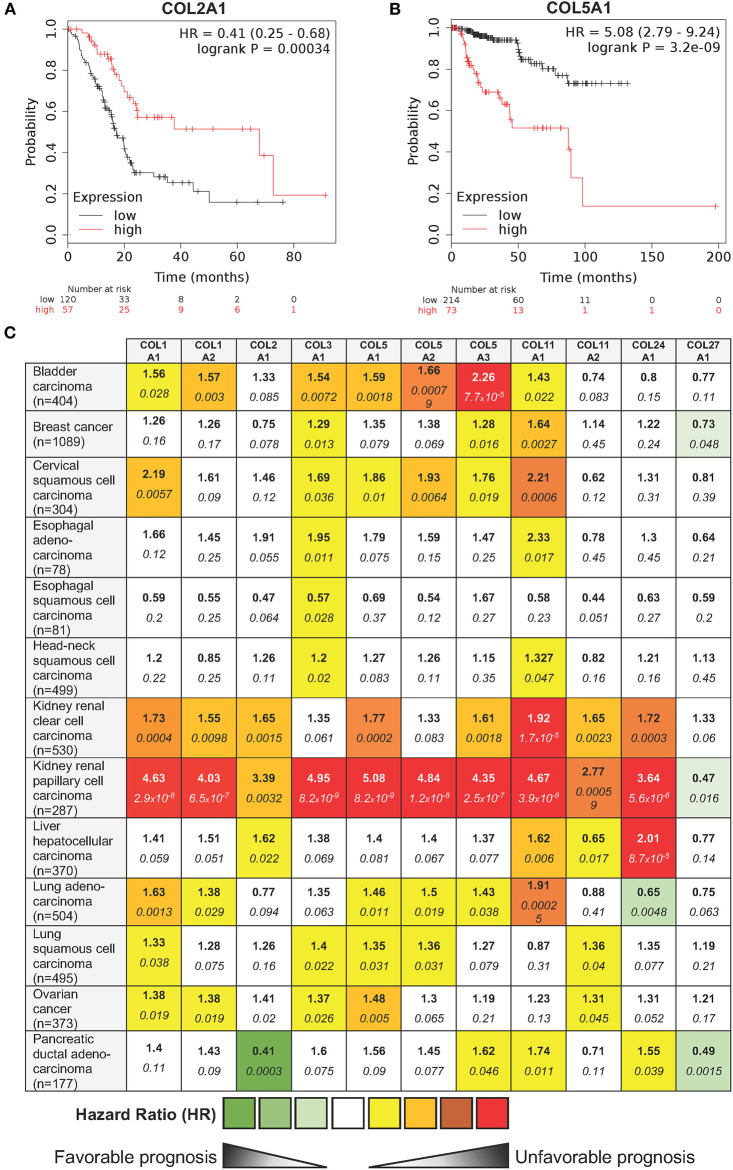
Influence of 11 fibrillar collagen gene expression on patient prognosis outcome in 13 different cancers. Hazard ratio (HR) and log-rank *p*-values were calculated using the pan-cancer RNA-seq Kaplan-Meier plotter (182). **(A)** Kaplan-Meier plot showing that patients with a high COL2A1 gene expression (red lines) have a higher overall survival than those with a low gene expression (black lines). **(B)** Kaplan-Meier plot showing that patients with a high COL5A1 gene expression (red lines) have a lower overall survival than those with a low gene expression (black lines). HR and 95% confidence interval are shown. Log-rank *P* < 0.05 was considered to indicate statistical significance. **(C)** Summary of HR (**bold**) and log-rank *p*-values (*italic*) for 11 fibrillar collagen genes in 13 different cancers. Collagen genes whose mRNA levels were significantly associated with patient's overall survival in a specific cancer were color coded according to the log-rank *p*-values and HR (unfavorable prognosis: yellow to red; favorable prognosis: light to dark green).

In order to complete this overview of the roles of fibrillar collagens in cancer, we explored *in silico*, whether the mRNA level of the different fibrillar collagens were connected with clinical outcome in human cancers. Patients were divided into two groups (low and high expression groups) according to the gene expression level in tumor tissue. Kaplan-Meier log rank analysis showed that a high expression level of a few collagens was associated with a significantly longer overall survival period in some cancers (e.g., COL2A1 in pancreatic ductal adenocarcinoma—Figure 4A). However, for most fibrillar collagens, high expression levels were linked with significantly shorter overall survival periods (e.g., COL5A1 in kidney renal papillary cell carcinomas—Figure 4B). The complete analysis of the prognostic value of the expression level of 11 fibrillar collagens on the survival of patients suffering from 13 different cancers is summarized in Figure 4C.

## Fibrillar Collagens, Aging and Cancer: A Dangerous Trio

The incidence of most cancers strongly increases with age and cancer represents the primary cause of death in population aged 60–79 years. The risk of having an invasive cancer in patients over 60 is more than 2-fold that of younger patients (183).

Many studies have shown that aging affects the normal cells of the TME. Among these stromal cells, fibroblasts and immune cells, which represent key actors in tumor progression and metastasis, are notably susceptible to this age-related impact (184). ECM integrity decreases substantially as we age. Age-related alterations in the physical features of the ECM comprise decreases in collagen density (185–187), ECM fiber thickness and area (188, 189).

Because of their long half-life, fibrillar collagens are subjected to post-translational changes during biological aging. These modifications include mineralization, accumulation of advanced glycation end-products (AGEs) (190), an increase in crosslinks level (191), and the reduction of glycosaminoglycans (GAGs), which impact fiber stability. These biochemical alterations change the structural organization of type I collagen (192–194). Such changes in structural properties of type I collagen affect its susceptibility to matrix metalloproteinases (MMPs)-mediated degradation (185, 195), and lead to a tissues stiffening. These structural alterations also influence the recognition of the collagen fibers by cell surface receptors such as discoidin domain receptors (DDR), which are sensitive to the structural organization of the collagen. *In vitro* studies have shown that aged type I collagen upregulates the proliferation of fibrosarcoma cells (196) and presents a reduced pro-apoptotic potential toward luminal breast cancer cells (197).

## Imaging Fibrillar Collagens

Several techniques can be used to image the architecture of collagen networks depending on the experimental conditions and the studied features. Histochemical (van Gieson staining, Picro-Sirius red, etc.) and immunohistochemical techniques are routinely used to stain fibrillar collagens (198–200). These techniques require additional fixation and preparation steps, which can alter the collagen structure of the samples, and hence are limited to *ex vivo* materials. Images of stained samples are obtained using bright-field, polarized and fluorescence microscopy techniques (199, 200). Laser scanning microscopes or multiphoton laser scanning microscopes have also been used to visualize stained tissues in three dimensions (198).

Transmission electron microscopy (TEM) and scanning electron microscopy (SEM) also allow to visualize collagens *ex vivo* and *in vitro*. The analyzed tissues must previously be washed, dehydrated, dried and sputter coated before imaging (103, 201, 202). While SEM and TEM provide highly detailed information on microstructure and nanostructure of collagens, respectively, the preparation steps may greatly alter the samples. SEM can be used to image morphology and arrangement of the collagen fibers but its three-dimensional imaging capability is limited (103). Besides these methods requiring a preparative step, non-disruptive techniques are also available. Among these techniques, confocal reflection microscopy (CRM) is a high resolution technique for imaging specimens which differ in refractive index from their surroundings or which possess a high reflectance (203). It is widely used to visualize polymers and biomaterials. A laser-scanning confocal microscope in reflection is able to detect variations of backscattered light intensity at the collagen-to-media interface for each sequential focal plane, resulting in the reconstruction of a three-dimensional image of the sample (204). CRM is readily applicable to dynamically follow living specimens. This technique can be combined with fluorescence confocal imaging without modifying the microscope hardware (76). While this visualization technique is mostly used *in vitro* to study the interactions between cells and ECM components (76, 194, 201, 203, 204), it also enables the *in vivo* visualization of epidermis and superficial dermis in real time, providing a tool for imaging skin lesions and helping skin cancer diagnosis (205–207). Excitation wavelength is proportional to the minimal resolution but decreasing it reduces the penetration depth (204). Hence, simultaneous excitation at 488 and 567 nm leads to better imaging than individual 488 and 567 nm excitations. In practice, a penetration depth of up to 100 μm is achieved for CRM on 3D collagen scaffolds, while resolution strongly declines after 30 μm.

Second harmonic generation (SHG) microscopy is a highly specific optical method of direct visualization of fibrillar collagens that can be carried out using most two-photon fluorescence microscopes (208). It allows the non-invasive assessment of the abundance and structure of fibrillar collagens with a high resolution and specificity. For those reasons, it represents the most widely used technique for the *in vivo* imaging of fibrillar collagens.

**Figure 5:**
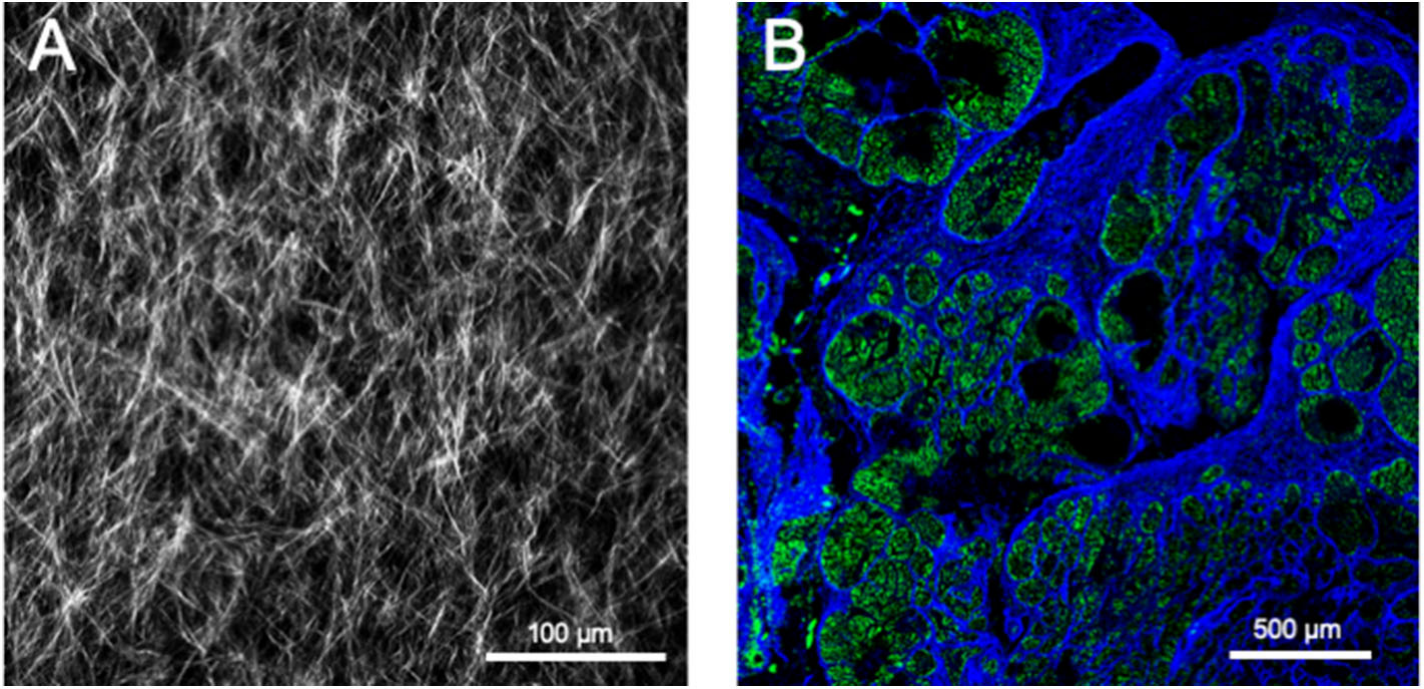
Second harmonic generation (SHG) image of fibrillar collagens. **(A)** SHG image of *in vitro* polymerized collagen I. **(B)** SHG of a mouse mammary PyMT tumor section. Collagen fibers appear in blue, while the cellular structures appear in green due to the autofluorescence of the sample.

SHG occurs when two photons interact with optically non-linear material and merge to generate a new photon with twice the energy and half the wavelength of the initial photons. Fibrillar collagen non-linear optical response, which results in a strong SHG signal, originates from its non-centrosymmetric triple helical molecular assemblies, which exhibit large hyperpolarizabilities (209, 210). This process is sensitive to the microscopic structure of the scattering material. Therefore, SHG emission directionality is influenced by the diameter of the collagen fibrils that are bundled into fibers, their spacing within the fiber, and the disorder in their packing (209, 211, 212). Among the different collagens, type I collagen has the most ordered structure and, hence, produces the strongest SHG signal (213). Maximum SHG resolution is higher than what is obtained using linear optical microscopy techniques and enables to image collagen fibrils (209, 214). SHG signal intensity is dependent of the fibril/fiber orientation (210). In particular, fibers which are perpendicular to the laser light polarization axis will result in a weak signal. SHG is also very low with fibers perpendicular to the imaging plane (due to the centrosymmetric structure of fiber cross section). It should also be emphasized that, due to resolution limitations, collagen fibrils with a diameter lower than 100 μm will not be detected. The SHG optical sectioning capability enables tissue imaging in three dimensions (215). SHG imaging is therefore a label-free, non-destructive, high resolution, sensitive and specific modality for visualizing the spatial distribution of fibrillar collagens *in vitro, ex vivo* (Figure 5), and *in vivo*. SHG images can be subsequently analyzed with specific image analysis tools such as CT-FIRE which can extract individual collagen fibers from images for a quantitative assessment of fiber metrics including fiber angle, fiber length, fiber straightness, and fiber width (216). In the context of cancer, SHG measurement has been used as an independent prognostic indicator of metastatic outcome in patients with estrogen receptor positive, lymph node-negative breast cancer as well as in stage I colon adenocarcinomas (217).

Other techniques can be performed to directly monitor collagen fibers. Small angle X-ray scattering (SAXS) characterizes collagen fibril structure and organization into fibers of whole samples (218, 219). It represents an efficient way for observing and investigating the structural organization of collagen fibrils including their orientation. Collagen fibril orientation can then be used to derive the collagen fiber orientation. When used in combination with Fourier transformed infrared (FTIR) microspectroscopy, which gives chemical information on submolecular bounds and functional groups, and unsupervised hierarchical cluster analysis, it can characterize, classify and map cell clusters in *ex vivo* tissue samples (219). Magnetic resonance imaging (MRI) can be used to study *ex vivo* and *in vivo* the three-dimensional organization and density of collagens (220, 221). Finally, real-time collagen imaging in *in vivo* models can also be achieved by using transgenic lines expressing fluorescent protein-tagged fibrillar collagen (222).

## Structural and Functional Features of Collagen-Binding Integrins

Fibrillar collagen receptors include different classes of molecules such as DDRs, mannose receptors, leucocyte receptor complex, as well as proteolytic enzymes. DDRs (DDR1 and DDR2) are tyrosine kinase receptors that become specifically activated by the native triple helix of fibrillar collagens I–III, followed by tyrosine autophosphorylation, receptor internalization and signaling (223, 224). Leucocyte receptor complex members like OSCAR (osteoclast associated receptor) and GPVI (glycoprotein VI) have been also shown to act as fibrillar collagen receptors and mediate processes such as osteoclastogenesis (225) and platelet activation and aggregation, respectively (226). The uPARAP/Endo180 mannose receptor has been shown to recognize fibrillar collagens such as collagen I, II, and V and acts as an endocytic receptor by modulating the collagen fragment internalization and its lysosomal degradation, playing a role in the fibrillar collagen turnover (227). In this context, proteolytic enzymes such as MMPs and cathepsins also recognize fibrillar collagens and mediate collagen degradation in the extracellular compartment (228). These different non-integrin collagen receptors have been shown to play key roles in specific hallmarks of cancer, including among others proliferation, apoptosis, drug resistance, inflammation, neo-angiogenesis and metastasis (227, 229).

**Figure 6:**
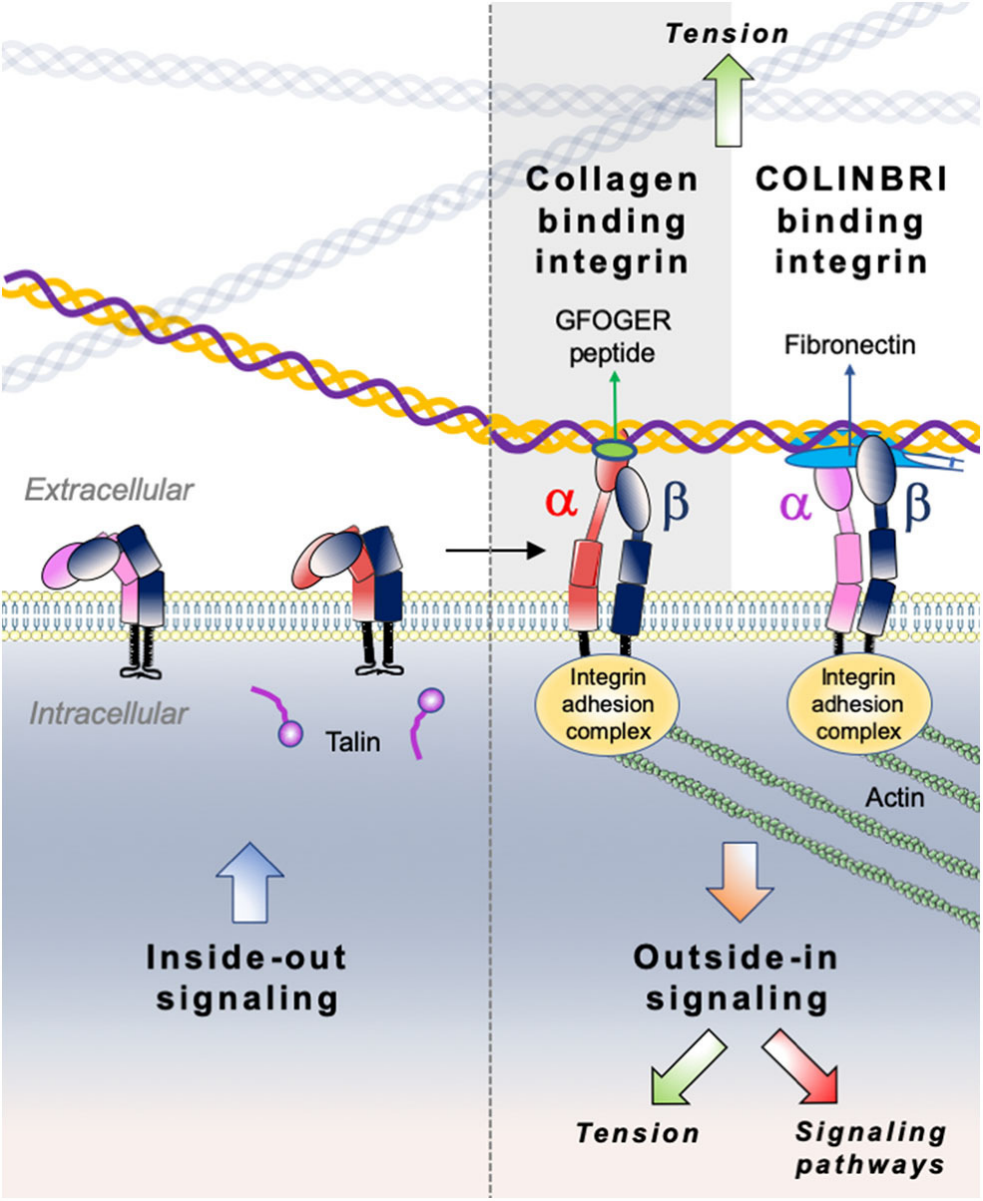
Illustration of direct (collagen-binding integrin-mediated) and indirect (COLINBRI-mediated) integrin heterodimers binding to collagen fibrils. Inactive integrins adopt a compact conformation in which the α- (red/purple) and β-subunit (black) are closely associated. Intracellular signals, culminating in the binding of talin to the β-subunit tail, lead to conformational changes that result in increased affinity for extracellular ligands. The primed integrin binds ligand, which represents the end-point of inside-out signaling. The binding of talin and ligand initiate focal contact formation. As the cytoskeleton matures, tension (green arrows) is generated on the integrin receptor across the cell membrane. The force applied to the integrin strengthens receptor-ligand binding and allows the formation of stable focal adhesions and the initiation of intracellular signaling cascades (red arrow), the end-point of outside-in signaling. In the direct cell-binding mechanism, collagen-binding integrins directly interact with the GFOGER sequence of fibrillar collagen to provide cell adhesion. In the indirect way, cell binding involves COLINBRIs like fibronectin represented here in blue. The COLINBRI molecule is anchored to collagen and provides cell attachment by interaction with the COLINBRI-binding integrins.

Integrins comprise a large family of matricellular receptors involved in the transduction of the bidirectional signaling between cells and the surrounding ECM. Each integrin consists of a cell surface heterodimer of α and β subunits non-covalently associated and composed by a large ectodomain, a transmembrane domain and a short cytoplasmic tail (Figure 6). The ECM ligand specificity of integrins is ascribed to the large ectodomain, while the cytoplasmic tail is known to bind several intracellular regulatory and cytoskeletal molecules. Integrin α and β subunits heterodimerize in the endoplasmic reticulum, undergo posttranslational modifications such as glycosylation in Golgi, and are subsequently transported in an inactive form at the cell surface where activated to interact with specific ECM ligands. Recognition of different ECM ligands by specific integrins triggers distinct intracellular signals, enabling cells to regulate their behavior in response to microenvironmental changes.

Among the 24 different integrin heterodimers (18 α subunits and 8 β subunits), collagen binding integrins include four different α subunits bound to a common β1 subunit: α1β1, α2β1, α10β1, and α11β1. Other classes of integrins include integrins presenting a Arg-Gly-Asp (RGD) motif, laminin binding integrins and leucocyte receptors. The members of collagen binding integrin subgroup recognize their fibrillar ligands either directly by using an inserted domain in their α subunit (αI domain) (230) or indirectly by a class of non-collagen bridging molecules—COLINBRI” (COLlagen INtegrin BRIdging) (231) (Figure 6).

Specific collagenous motifs are recognized by integrin αI domains, the major functional motif being the GFOGER (O = 4-hydroxyproline) sequence in the triple-helical conformation. The binding of this motif occurs in a metal-dependent manner via a Metal Ion-Dependent Adhesion Site (MIDAS) which coordinates a divalent cation Mg^2+^ and is highly conserved in all four collagen binding integrins (232). Other motifs with a GxOGER sequence (x = hydrophobic residue) include GROGER, GLOGER, GMOGER, and occur at specific loci within the D-periods of fibrillary collagens. A description of all known integrin recognition motifs for fibrillar collagens has been previously addressed by Hamaia and Farndale (233). Previous studies reported that α1 and α10 integrins poorly bind to the fibrillar form of collagens, when compared to their α2 and α11 counterparts, and rather favor the binding to the native monomeric form of other types of collagens such as network-forming (collagen IV and VI) or FACIT collagens (collagen IX) (231). It has been demonstrated that integrin α1 prefers to bind to the monomeric form of collagen I rather than to its mature fibrillar form (234) and also binds with a higher affinity to the collagen IV (a basement membrane collagen) (235). Likewise, integrin α10 displays a higher affinity for network-forming collagen IV and VI when compared to the fibrillar collagens I-III (236). Higher fibrillar collagen specificity was however demonstrated for the α2 and α11 integrin I domains. These integrins prefer the binding of mature fibrillar collagen forms mostly through the GFOGER motif. Along with the binding of collagenous ligands, these integrins show also specificity for other ECM components such as laminins, proteoglycans, and tenascins. A detailed ligand specificity of collagen binding integrins is described in Table 3.

**Table 3:** Specificity of collagen-binding integrins.

**Integrin subunit**	**Collagen specificity**	**Motifs**	**Other ECM ligands**	**References**
α1	Collagen I, II, III, IV, V, VI, IX, XVI and XVIII Highest affinity for collagen IV	GFOGER (collagen I, II, IV) GVOGEA (collagen II) GLOGEN (collagen III)	Laminins, galectins, arresten and semaphorin 7A	(231–233, 237, 238)
α2	Collagen I, II, III, IV, V and XI	GFOGER (collagen I, II, IV) GMOGER	Laminins, Tenascin C, decorin, endorepellin, perlecan, and chondroadherin	(231–233, 239, 240)
α10	Collagen I, II, IV, VI Highest affinity for collagen IV and VI	GFOGER (Collagen I, II, IV)	ND	(233, 236, 241)
α11	Collagen I, IV, V, IX Highest for collagen I	GFOGER (Collagen I, II, IV)	ND	(231, 232, 242, 243)

Most of the functional motifs in the collagen triple helix contain hydroxylated proline residues. Along with its critical role in the stabilization of the collagen triple helix and fibril formation, hydroxylation of proline in position Y of the collagen triplet sequence -G-X-Y- has also an important role in the recognition of functional sites by the collagen receptors. It has been reported that α2 integrin binding to the GFPGER motif (P = 4-proline) is weaker than its binding to the consensus GFOGER motif, suggesting a higher avidity of integrin receptors for hydroxyproline-containing motifs of collagens (244). Furthermore, a recent study reported that absence of hydroxyproline residues in the GFOGER motif strongly impairs the avidity of α1 and α11 integrins for the collagen molecule and with a less prominent difference for the α2 integrin (245). This study revealed that absence of proline hydroxylation in collagen can affect integrin binding not only by the structural destabilization of the triple helix, but also by a direct mechanism, in which the residue Arg-218 in the α1I integrin domain directly interacts with the hydroxyproline residue in the integrin-binding motif of collagen.

Indirect binding of fibrillar collagens may involve collagen-binding integrins but also RGD integrins or leucocyte receptors. COLINBRIs have been described as prototypical ECM molecules being able to bridge between integrins and fibrillar collagens. Such molecules include among others fibronectin, vitronectin, periostin, and small leucine-rich proteoglycan/protein (SLRP) family members (231). These proteins show binding sites for specific fibrillar collagens, as well as several integrin-recognizing sites. They also play a role in collagen fibrillogenesis, deposition, and modulation of integrin affinity for collagenous ligands. An example of such a COLINBRI molecule is fibronectin, which displays a binding domain for the collagen Iα1 chain in its N-terminal part and several binding sites for RGD-binding integrins (α5β1 or αvβ3 integrins) and leucocyte receptors (α4β1 or α4β7 integrins). Previous studies have reported the reciprocal mechanoregulation between the two ECM molecules and the active role of fibronectin in the collagen initial fibril-formation and assembly (246). In this context, collagen-binding integrins have also been reported to interact with COLINBRI-like molecules, particularly with SLRPs. Decorin interacts with α2β1 integrin and allosterically modulates its collagen I-binding activity in angiogenic endothelial cells, but not quiescent cells, thus promoting a migratory phenotype (247). Conversely, lumican, another SLRP involved in collagen type I fibrillogenesis, displays an inhibitory effect on melanoma cell migration modulated by the interaction of its proteic core with the activated I domain of the α2 integrin subunit (248). For a detailed comparison of integrin and non-integrin collagen receptor binding sites, we refer the reader to Zeltz et al. (231) and Zhu et al. (249).

## Mechanistic Insights into Collagen-Binding Integrin Signaling

Collagen-binding integrins, similarly to the other type of integrins operate as bidirectional signaling receptors upon a biochemical or mechanical activation. The two directions of integrin signaling have different biological consequences and involve distinct conformational states. In the traditional “**outside-in**” signaling, integrins act as membrane receptors in transmitting information from the surrounding environment into cells (Figure 6). This signaling refers to multivalent integrin-ligand binding, integrin conformational switch from an inactive low avidity state to a high avidity state, subsequent clustering and association with actin cytoskeleton (250). During “**inside–out**” signaling, talin, an intracellular activator, binds to the cytoplasmic tail of integrin β-subunit, inducing conformational changes that increase the affinity for extracellular ligands (251). Although conceptually different, the “outside-in” and “inside-out” integrin signaling processes are often complementary and closely linked both leading to regulation of cell polarity and cytoskeleton assembly, of gene expression, cell survival and motility. It is worth noting, that most of the previously described mechanistic studies on integrin signaling were performed on RGD binding integrins, as well as on α1β1 and α2β1 collagen-binding integrins, while integrin α10β1 and α11β1 signaling is still poorly explored. Furthermore, the specificity of integrin signaling relies on multiple factors, including heterodimer subtype, cellular context, ECM organization and ligand recognition. Analogously to other integrin subtypes, the signaling responses of collagen binding integrins are mostly mediated by the common β1 subunit and are not specific to the αβ heterodimer. Only a few studies reported the implication of the α cytoplasmic domain in the cellular signaling of collagen binding integrins. We therefore described below a general integrin signaling pathway applied to most integrins and highlight relevant studies which describe distinct signaling events for collagen binding integrins.

Integrin signaling requires the assembly of a dynamic multiprotein machinery around their cytoplasmic tails, called “adhesome.” It was reported that integrin adhesome comprises a network of at least 156 components linked via 690 different interactions (252). These components are classified into several functional groups, which comprise 25 adaptor proteins, 24 cytoskeletal molecules, nine actin-binding proteins, 18 tyrosine and serine/threonine kinases, 12 tyrosine and serine/threonine protein phosphatases, seven transmembrane receptors, six adhesion proteins, eight GTPase-activating proteins (GAPs), eight guanine nucleotide exchange factors (GEFs), five GTPases, and 32 other components (252). Integrin connection to the cytoskeletal machinery is mediated by integrin adhesion complexes (IACs)—macromolecular complexes which transduce biochemical and mechanical signals from the microenvironment into biological responses (253). The formation of several major types of IACs has been reported, including focal complexes, focal adhesions and fibrillar adhesions, which play a central role in cell adhesion and migration (253). Most of the IAC proteomic analyses were performed on complexes isolated from fibronectin-attached cells, while still little is known about the adhesome complexes related to collagen binding integrins. These studies identified around 60 core proteins within IACs which constitute the cell adhesion machinery and link integrins bound to their ECM ligands to the cytoskeleton (254). It is plausible to assume that IACs associated to collagen binding integrins share a great similarity with those derived from fibronectin-attached integrins, as their have a common β1 subunit.

Integrin clustering refers to the interaction of integrin αβ heterodimers to form hetero-oligomers at cell surface. This event is relevant for “outside-in” signaling of integrins, including mechano-transduction processes and integrin recycling (251, 255). Upon clustering, integrins associate with a cytoskeletal signaling complex that promotes the assembly of actin filaments into large stress fibers (251). The resulting intracellular structures formed by integrins and cytoskeletal proteins are known as “focal adhesions” and “focal complexes.” Early steps of integrin signaling involve interactions with tyrosine kinases such as focal adhesion kinase (FAK), Src-kinases, abelson murine leukemia viral oncogene homolog 1 (Abl) and integrin-linked kinase (ILK), scaffold molecules such as p130CRK-associated substrate (p130CAS) and cytoskeletal proteins such as talin and kindlin. In the canonical integrin pathway, the active FAK/Src complex interacts with p130CAS and paxillin that in turn recruit Crk adaptor molecule leading to activation of several downstream molecules, including ras-related C3 botulinum toxin substrate 1 (Rac1), serine/threonine-protein kinase PAK 1, and c-Jun N-terminal kinase (JNK). The FAK/Src complex can also recruit other adaptor molecules leading to activation of major signaling pathways such as PI3K, RhoGTP-ases, p38, Erk, and phospholipase gamma (PLCG) pathways (256). Several studies have reported the implication of collagen binding integrins in multiple pathways mentioned above and mainly for α1β1 and α2β1 integrins. For example, binding of collagen type I by integrin α1β1 has been shown to sustain mesangial cell spreading via the activation of Erk1/2 pathway (257). Collagen binding of integrin α2β1 through the GFOGER motif has been shown to mediate an “outside-in” signaling and to promote platelet spreading via activation of Src, FAK, and PLCG2 pathways (258). Similarly, integrin α2β1-mediated adhesion of platelets to type I collagen or GFOGER peptide has been shown to induce a downstream Pyk2 activation and PI3Kβ and Akt phosphorylation (259). Platelets adhesion to the monomeric collagen type I via α2β1 integrin was also shown to trigger the activation of PLCγ2 downstream signaling via Src kinases and Rac GTPases (260). Interestingly, both α1 and α2 collagen binding integrins have been shown to be involved in the production of reactive oxygen species (ROS), either by negatively regulating Rac1 activation and collagen synthesis through a crosstalk with the epidermal growth factor receptor (EGFR) (261), or by p38 MAPK phosphorylation and regulation of cell cycle (262). Furthermore, α11β1 integrin has been also shown to be required for an efficient collagen remodeling in wound healing via the non-canonical TGF-β1-dependent JNK signaling (263).

“Inside-out” signaling of integrins involves interactions with intracellular molecules at the cytoplasmic tails, which regulate integrin conformation. These interactions mainly occur at the cytoplasmic domain of the β integrin subunit. Talins and kindlins play a key role in this process. The binding of adaptor protein talin to the cytoplasmic tail of integrin β-subunit is a critical event in affinity activation of integrins (264). Talin association triggers integrin activation through the disruption of inhibitory interactions between α- and β-subunit cytoplasmic tails with subsequent transition from the bent to the extended conformation and increase in integrin affinity for extracellular ligands (255). Another major regulator of inside-out signaling, kindlin binds to the cytoplasmic tail of integrin β-subunit, supports integrin activation and recruits paxillin to the nascent focal adhesions, which in turn promotes the formation of membrane protrusions and therefore cell migration (265). In this context, talin was shown to be required for α2β1 integrin-mediated platelet adhesion to collagen type I and to promote platelet aggregation (266). Interestingly, another study reported that the interaction between integrin α2β1 in a non-activated conformation and collagen type I results in the activation of FAK in a talin-independent manner and requires the protein kinase C (267). While most of these interactions involve the β1 cytoplasmic tail, some studies also reported the role of α1 and α2 subunits in the integrin downstream signaling. Notably, SHARPIN, an inactivator of talin and kindlin recruitment to β subunits, has been found to bind α1 and α2 tails, rather than β1 cytoplasmic domain (268). The T-cell protein tyrosine phosphatase (TCPTP) has been also found to bind the cytoplasmic tail of α1 integrin upon cell adhesion to collagen and to negatively regulate EGFR signaling (269). Similarly, it has also been reported that α2 cytoplasmic tail is involved in the downstream activation of p38 signaling and upregulation of collagen gene transcription (270). The distinct signaling events induced by the different collagen binding integrin α subunits is remarkably highlighted by the opposite effect that these subunits have on collagen synthesis. Indeed, while α1β1 integrin is a known repressor of collagen type I synthesis (271), α2β1 integrin promotes its expression (270). Additionally, several studies have reported a link between the ligand-induced activation of collagen binding integrins and the production of collagen remodeling proteinases such as MMPs (272–274).

Collagen-binding integrins have been also reported to crosstalk with receptor tyrosine kinases (RTKs) and mediate many cellular functions, including signaling, ligand recognition, and RTK endocytosis and trafficking. Multiple studies have reported the spatial coexistence between integrins and RTKs at cell surface with a synergistic signaling between the two receptors mainly related to the “outside-in” signaling of integrins (275). The interaction between the two partners was reported to be ligand-dependent or independent. A detailed description of these interactions was addressed by previous reviews (275–277). In the context of collagen binding integrins, a crosstalk between EGFR and α1β1 or α2β1 integrins has been reported to regulate the downstream signaling and trafficking of this tyrosine kinase receptor (269, 278). The cooperation between HGFR and α2β1 integrin has been shown to regulate the innate immune response upon mast cell adhesion to collagen type I (279). Furthermore, we have recently shown that integrin α11 associates to PDGFRβ in cancer associated fibroblasts and activates its JNK downstream signaling (280).

DDRs can also positively and negatively regulate collagen-binding integrin-mediated signal transduction and cell adhesion: DDR-mediated signaling can directly affect the activity of integrins but it can also converge with integrin-triggered pathways to regulate cellular functions, whereby each receptor engages its own downstream pathway (281).

## Collagen-Binding Integrins in Cancer

Integrin expression patterns undergo major changes during tumor progression and metastasis leading to significant alterations in the phenotype of both cancer and stromal cells. In cancer cells, integrins have a key role in sustaining cell proliferation and invasion, evading tumor suppressors and cell death and promoting the EMT (282). Stromal integrins, on the other hand, are implicated in processes such as tumor angiogenesis, desmoplastic reaction and metastasis by modulating cell adhesion and invasion, and ECM remodeling. In view of their bidirectional signaling, integrins provide spatially restricted communication between cells and the surrounding microenvironment and act as key mechanosensing elements and RTK co-partners to modulate biological processes essential for tumor cell survival. Normal cells rely on integrin-mediated cell adhesion to ECM components to proliferate and survive. Defects in cell ability to attach to ECM are associated with impaired pro-survival integrin-dependent signaling pathways including PI3K/Akt, MAPK, FAK, NF-κB, leading to anoikis (283). Integrins modulate all steps of the metastatic process, from invasion from the primary tumor and intravasation into the bloodstream, to colonization of the secondary sites. Distinct integrin-ligand binding combinations act as drivers of organ-specificity colonization of tumor cells and the subsequent cell survival and adaptation to the newly acquired microenvironment. The desmoplastic reaction has been linked to high expression levels of integrins in metastatic cancers (284). Being key modulators of ECM, tumor and stromal integrins regulate matrix composition in order to control cell adhesion and invasion. Along with the aforementioned processes, integrins also contribute to tumor regulation through angiogenesis, metabolic reprogramming, evasion of immune destruction and acquisition of drug resistance.

Several mechanisms have been found to be involved in collagen-binding integrin regulation of tumor growth and metastasis. In the following section, we will discuss each of the four collagen-binding integrins, their expression profile and implication in pathological conditions with a special focus on cancer disease.

**α1β1** integrin is widely expressed in normal tissues, particularly in the mesenchyme, vascular and immune system with limited expression in the epithelium (285). As illustrated in Table 3, this integrin can bind many types of collagens, including collagen I, II, III, IV, V, VI, IX, XVI, and XVIII, as well as laminins, galectins, arresten, and semaphorin 7A (232). As previously mentioned, α1β1 integrin is mostly detected in connective tissues and it exhibits a higher affinity for collagen IV when compared to collagen I (235). Integrin α1β1 functions include cell adhesion and survival, collagen synthesis and MMP secretion. This receptor has been associated with pathological conditions such as osteoporosis, renal injury, rheumatoid arthritis, psoriasis, as well as cancer (285). Integrin α1 mutant mice are viable and fertile with no marked phenotype (286). Integrin α1 adult mice exhibit however a mild decrease in body weight, hypocellular dermis (287) and aged animals display different phenotypes, including osteoarthritis (288), and retinal degeneration (289). *In vitro*, integrin α1-deficient cells display adhesion alteration when cultured on collagen I and IV, as well as failure to recruit and activate Shc adaptor molecule with consequent reduction in cell proliferation and survival (287). When challenged to different pathological conditions, α1-mutant mice present reduced psoriasis (290), accelerated aging-dependent osteoarthritis (288), diminished bone fracture healing (291), severe hepatic insulin resistance and lower hepatic fat accumulation upon a high fat diet (292), and increased glomerulosclerosis in diabetic mice (293) (Table 4).

**Table 4:** Phenotypical consequences of collagen-binding integrin knockouts in mice.

**Integrin subunit**	**Expression**	**Knockout phenotype**	**Pathologically challenged knockout phenotype**	**Cancer phenotype**
α1	Endothelial cells, fibroblasts, pericytes, mesangial cells, bone marrow mesenchymal stem cells, hepatic stellate cells, chondrocytes, neural cells, Schwann cells, immune cells (285, 286)	No pronounced phenotype (286) moderate decrease in body weight, hypocellular dermis (287) aged-related osteoarthritis (288), retinal degeneration (289)	Reduced psoriasis (290) and inflammation (129), accelerated aging-dependent osteoarthritis (288), reduced bone fracture healing (291), increased glomerulosclerosis in diabetic mice (293), hepatic insulin resistance and decreased hepatic fat accumulation on a high fat diet (292)	Reduced tumor number and size, reduced angiogenesis and proliferation, increased apoptosis in models of non-small cell lung carcinoma α1-null mice (294, 295); overexpression in colorectal cancers and correlation with Myc oncogene; reduced angiogenesis, increased necrosis and low mitotic index in a xenograft model of colorectal cancers (296); reduced angiogenesis in an experimental breast cancer model (297); correlation with a poor patient outcome in melanoma (298, 299); gain of expression in oral squamous cell carcinomas, broncho-alveolar and gastric carcinomas (300–302)
α2	Epithelial cells, platelets/megakaryocytes, endothelial cells fibroblasts (303, 304)	Moderate phenotype in mammary gland morphogenesis (305, 306), adhesion defects for isolated platelets on collagen I (307), reduced age-related osteoporosis (308)	Increased neovascularization in wound healing (309), defects in age-related bone loss (308), decreased immune response to peritoneal Listera infection and reduced thrombi formation (239), decreased glomerulosclerosis upon renal injury (310), reduced inflammation and cartilage deterioration in rheumatoid arthritis (311), reduced inflammatory bowel disease (65)	Metastasis suppressor in the MMTV-neu mouse breast cancer model and correlation with patient outcome in breast and prostate cancers (312); impaired breast cancer metastasis in a xenograft model of MCF-7 cells interacting with platelets (313); reduced MMP13 expression by metastasizing cells to the bones in an experimental model of MDA-MB-231 breast cancer cells (314); impaired tumor angiogenesis in a melanoma xenograft model in a double knockout model (66); co-localization with E-cadherin and N-cadherin at tumor cell-cell contacts in primary and metastatic samples of melanoma (315); selective metastasis to the bones in prostate cancer (316); implication in doxorubicin-induced drug resistance in leukemia (317, 318); implication in other types of cancers, including pancreatic, colorectal, gastric and lung cancers, oral squamous cell carcinoma and T-cell acute lymphoblastic leukemia (319)
α10	Chondrocytes, mesenchymal stem cells, junctional fibroblasts (241, 320)	Mild chondrodysplasia (321), canine chondrodysplasia and limb dwarfism (322)	ND	Upregulation in malignant melanoma (323); overexpression in glioblastoma (324); high expression correlated with disease-specific death and distant metastasis in high-grade myxofibrosarcoma (325)
α11	Fibroblasts (cancer associated fibroblasts, myofibroblasts), mesenchymal stem cells (326–328)	Dwarfism, altered incisor tooth eruption, increased mortality and decreased IGF-1 serum levels (327, 329, 330)	Reduced wound strength and deposition of granulation tissue in wound healing (263), reduced cardiac fibrosis and left ventricular hypertrophy (331)	Decreased tumorigenicity of cancer cells (328) and matrix stiffness (332) in a challenged xenograft model of lung cancer; correlation with tumor progression and postoperative recurrence in non-small cell lung cancer (333); association with a pro-tumorigenic PDGFRβ-positive subset of cancer associated fibroblasts and correlation with a poor clinical outcome in breast cancer (280); correlation with aggressive phenotypes of breast cancer (334); decreased intratumoral interstitial fluid pressure and collagen structure in a challenged mouse tumor model (335)

In the context of cancer, integrin α1β1 is still poorly explored. Only a few studies reported its role in tumor progression and metastasis, mostly in non-small cell lung carcinoma and colorectal cancer. In an orthotopic model of non-small cell lung carcinoma in α1-null mice with increased MMP9 levels, a decreased number, size and vascularization of primary tumors and metastases was observed, highlighting the proangiogenic features of this integrin (294). Similarly, in another study of a spontaneous non-small cell lung carcinoma mouse model in KrasLA2/α1-null mice, tumors appeared smaller, less angiogenic, with reduced proliferation and increased apoptosis (295). Overexpression of α1β1 integrin has been reported in 65% of colorectal cancers and correlates for more than 70% with Myc oncogene expression (336, 337). Furthermore, in a xenograft model of colorectal cancers, α1-deficient tumors displayed extensive necrosis, low mitotic index and reduced angiogenesis (296). Reduced angiogenesis in absence of α1β1 integrin was also reported in an experimental breast cancer model (297). Some melanoma studies also reported a link between high expression of α1β1 integrin and a poor patient outcome (298, 299). Oral squamous cell carcinomas, broncho-alveolar and gastric carcinomas were also reported to display a gain of α1β1 integrin expression (300–302).

**α2β1** integrin is mostly expressed by normal epithelial cells, fibroblasts and platelets/megakaryocytes, and depending on the differentiation state also in T lymphocytes and endothelial cells (239). It preferentially binds to fibrillar collagen types I, II, III, V, and XI. It also binds to non-collagenous ligands such as laminins, tenascin C, decorin, endorepellin, and chondroadherin (232). One of the main functions of integrin α2β1 is the adhesion-mediated survival particularly in platelets, where this integrin constitutes the most abundant collagen receptor. It is also a major regulator of cell motility, mainly via p38 MAPK pathway activation (239). Genetic alteration of integrin α2β1 expression has been detected in pathological conditions such as hemostasis, thrombosis, fibrosis, and immune response. The mutant mice for integrin α2 integrin subunit present a mild phenotype in the mammary gland branching morphogenesis defect (305, 306) adhesion defects for platelets, fibroblasts and keratinocytes when tested for collagen I (307), and a reduced age-related bone deterioration (308). Pathologically challenged mice display, reduced wound healing with increased neovascularization (309), defects in age related bone degradation due to the over-expression of collagen I (308), decreased innate immune response to Listera infection and reduced thrombi formation (239), decreased glomerulosclerosis and collagen deposition after renal injury (310), and reduced joint inflammation and cartilage destruction in rheumatoid arthritis (311).

In cancer, α2β1 integrin was extensively studied in the context of angiogenesis. In the MMTV-neu spontaneous mouse model of breast cancer, α2β1 integrin acts as a metastasis suppressor by inhibiting tumor cell intravasation without altering tumor development or growth (312). The same study provided evidence for a correlation between α2β1 integrin expression and estrogen positivity in breast cancer patients and that the decreased α2β1 integrin levels predict metastasis and decreased survival in prostate and breast cancer patients. Conversely, in another recent study of a xenograft model of MCF-7 cells interacting with platelets, α2 downregulation prevented breast cancer metastasis (313). In an experimental model of breast cancer metastasis, the MMP13 expression observed in MDA-MB-231 metastasizing cells to the bones was induced by collagen type I through integrin α1β1 and α2β1-mediated p38 MAPK pathway (314). The implication of α2β1 integrin, as well as α1β1 integrin was further emphasized by a melanoma xenograft model in a double knockout model, in which absence of both integrins impaired tumor angiogenesis (66). In another melanoma study, α2β1 integrin co-localized with E-cadherin and N-cadherin at tumor cell-cell contacts in both primary and metastatic samples (315). Disturbance of these adhesive networks impaired tumor growth in a xenograft model and N-cadherin downregulation prevented α2β1 integrin-mediated tumor cell invasion on collagen type I matrix. In prostate cancer, α2β1 integrin contributes to a selective metastasis to the bone, rather than to other sites in patient samples and downregulation of this integrin impairs the adhesion and migration of prostate cancer cells toward collagen type I within the bone (316). Implication of α2β1 integrin in tumor progression and metastasis was reported in several other types of cancers, including pancreatic, colorectal, gastric and lung cancers, oral squamous cell carcinoma and T-cell acute lymphoblastic leukemia, which were previously reviewed in (319). Lastly, it has been reported that α2β1 integrin and collagen I participate to doxorubicin-induced drug resistance in leukemia by either protecting leukemia cells from apoptosis through MAPK/ERK pathway activation (317), or by decreasing the DNA damage through the inhibition of Rac1 activation (318).

**α10β1** integrin expression is mostly restricted to the cartilaginous tissue and this integrin is considered as a phenotypic marker for chondrocyte differentiation. Integrin α10β1 is a receptor for fibril-forming collagen type II, for network-forming collagen IV and beaded-filaments forming VI (321). It has a lower affinity for other types of fibrillar collagens. Integrin α10β1 is implicated in chondrocyte differentiation potency and is associated to an increase of collagen type II synthesis (338). The major physiological role of α10β1 integrin concerns the skeletal development, as this receptor is mainly expressed in the cartilage. Indeed, α10β1 integrin ablation in mouse models results in mild chondrodysplasia with altered chondrocyte functionality (321). Integrin α10-deficiency is associated to changes in chondrocyte morphology, increased apoptosis and reduced proliferation, resulting in a mild growth retardation (339). Furthermore, a previous study reported that canine chondrodysplasia is associated with a naturally occurring mutation of α10β1 integrin gene, and the observed limb dwarfism resembled the phenotype detected in the α1-null mouse model (322). The implication of this integrin in other pathological conditions is poorly documented.

In cancer, the role of α10β1 integrin remains poorly explored. A previous study reported the upregulation of α10β1 integrin in malignant melanoma when compared to primary melanocytes (323). In a recent study, this integrin emerged as a potential therapeutic target for treatment of glioblastoma (324). This study revealed that α10β1 integrin is overexpressed in glioblastoma patient tissues and cells when compared to normal brain tissues, and high α10 expression was associated to increased proliferation and migration. Interestingly, α10β1 integrin was correlated to disease-specific death and distant metastasis in a cohort of 64 primary high-grade myxofibrosarcomas (325). The authors demonstrated that α10β1 integrin promotes tumor cell survival through activation of TRIO-RAC-RICTOR-mTOR signaling, and that this pathway constitutes a promising targeted therapeutic strategy for patients with high-risk myxofibrosarcoma.

**α11β1** integrin expression is primarily confined to mesenchymal-like cells during pathological conditions including fibrosis, wound healing and cancer. Integrin α11 is the latest integrin family member to be identified. α11β1 integrin binds with high affinity collagen type I and contains the I domain, which recognizes the triple-helical GFOGER motif sequence (340). The highest α11 transcript levels were detected in embryos and human adult uterus, heart and periodontal ligament. A complete characterization of α11 expression in human adult tissues has not yet been performed. While the other collagen binding integrins α1 and α2 are expressed in a several cell types, α11 expression appears to be restricted to distinct cell subsets of mesenchymal origin. Generally, it is considered that α11 integrin is expressed *in vitro* by mesenchymal derived cells, and *in vivo* by fibroblasts at sites of highly organized collagen structures (230). Integrin α11 expression is promoted by TGFβ (341) and by the mechanical stiffness of the environment in a mechanism involving an autocrine loop of Activin (326). Integrin α11 is a major collagen receptor on fibroblastic cells and *in vitro* studies revealed that this integrin plays a key role in the adhesion and motility of these cells (242). Additionally, α11 integrin plays a critical role in collagen reorganization and remodeling. Several studies reported that *in vitro* integrin α11-deficiency impacts contraction of collagen matrices (273, 329). Furthermore, integrin α11 was also reported to be also implicated in TGFβ-induced myofibroblast differentiation (326). Interestingly, a recent study investigated the contribution of α11 integrin cytoplasmic tail to cell proliferation and invasion via a FAK/ERK-dependent activation (342).

The α11-deficient mice are viable and fertile but display the following defects: dwarfism, altered teeth, increased mortality, and decreased IGF-1 serum levels (327, 329). Dwarfism of α11-deficient mice does not seem to be related to structural defects in forming cartilage or bone, but it is rather due to the tooth alteration and IGF-1 serum levels in these mice. Indeed, a strong malnutrition of α11-deficient mice was observed due to a late incisor eruption and altered tooth shape (327). However, further investigations revealed that α11-deficient mice are already smaller at birth, before the incisor eruption effect could impact the body size, suggesting that other mechanisms could be involved in the acquired phenotype. Accordingly, IGF1 is a growth factor with a crucial role in growth control and bone mineralization. Indeed, low hepatic IGF1 production was detected in α11-deficient mice, implying that dwarfism observed in these mice may also be induced by the severely diminished IGF1 serum levels (330).

Only a limited number of studies have investigated the function of integrin α11 in pathological conditions. In wound healing, it has been reported that upregulated integrin α11 promotes wound strength and deposition of granulation tissue through a TGFβ-dependent JNK signaling (263). Overexpression of integrin α11 has been also linked to left ventricular hypertrophy and cardiac fibrosis, as a result of soluble factor secretion and increase collagen deposition in the heart (331). Recent studies have extensively investigated integrin α11 in the context of cancer associated fibroblasts. In lung cancer, stromal integrin α11 increases the tumorigenicity of cancer cells in xenograft models by modulating both IGF-2 production (328) and matrix stiffness (332). Likewise, another study reported integrin α11 association with tumor progression and postoperative recurrence in non-small cell lung cancer (333). We have recently demonstrated that integrin α11 identifies a PDGFRβ-positive subset of cancer associated fibroblasts displaying pro-tumorigenic features in breast cancer. Our study revealed that high stromal integrin α11/PDGFRβ expression is correlated with a poorer clinical outcome in breast cancer patients and that this integrin harnesses the PDGFRβ/JNK signaling pathway to promote the invasion of cancer cells (280). The implication of stromal α11β1 integrin in breast cancer was further investigated in another recent study, which emphasized the correlation of this integrin expression with aggressive phenotypes of breast cancer (334). Furthermore, an additional report highlighted the contribution of integrin α11 to breast cancer progression by regulating the intratumoral interstitial fluid pressure and collagen structure (335). Interestingly, a recent study analyzed the expression of α11β1 integrin in a panel of 14 different types of cancers and identified that this integrin is expressed by subset of non-pericyte-derived cancer associated fibroblasts and constitutes an important receptor for collagen remodeling. Integrin α11 contribution to other neoplastic malignancies and metastatic dissemination has not been yet documented. Its restricted expression, confined to a distinct mesenchymal cell subset, makes integrin α11 a good candidate for targeting the stromal compartment in cancer.

## Conclusions and Perspectives

Even if the hallmarks of cancer are driven by oncogenic mutations, most of them are modulated by the biochemical and biomechanical properties of the ECM that surrounds the tumor. By using different cell surface receptors, including integrins, cancer, and stromal cells are able to both impact and be impacted by ECM components in general and, more specifically, fibrillar collagens. In agreement with this concept, collagen fibers should no longer be viewed as passive scaffolds but as complex and constantly evolving signaling hubs that modulate a multitude of cellular functions.

It is now well-accepted that cancer should be viewed as a disease affecting the entire tissue rather than single cells. Indeed, tumors evolve in complex, dynamic, and functionally diverse TMEs. The diversity of TMEs depends on several factors including among others the tissue wherein the tumor develops, the stage of tumor evolution, the age of the patient. In contrast to cancer cells, the different cell populations which compose the TME are genetically stable, making them attractive targets for the development of innovative therapies with a low risk of development of treatment resistance. Furthermore, the efficacy of several standard chemotherapies and targeted agents is modulated by the TME, supporting the relevance of combining cancer cell-targeting agents with TME-directed therapies. However, issues related to cancer cell heterogeneity hold true for the TME. In that respect, a high degree of variability has been observed in ECM deposition and stiffness in a single tumor (343). Heterogeneity of the ECM could unravel the lack of success of the clinical trials of therapeutics targeting this feature of tumor development (343). We should also keep in mind that specific ECM components might have opposing roles during tumor progression in different cancers, suggesting that the impact of the ECM on the hallmarks of cancer cannot be broadly extrapolated to all cancer types.

It is worth noting that fibrillar collagens-derived proteolytic fragments are released in the blood stream where they represent easily accessible biomarkers to monitor cancer progression (140, 344–346).

While many questions related to how the biochemical and biomechanical properties of fibrillar collagens determine tumor progression remain unanswered, the potential for these ECM components to be effective markers and/or targets in treating cancer patients remains very promising.

## Author Contributions

IB, IP, TL, AN, and EM drafted the first version of the manuscript and IB, IP, and EM the figures and tables. All authors have critically reviewed and approved the manuscript.

## Conflict of Interest

The authors declare that the research was conducted in the absence of any commercial or financial relationships that could be construed as a potential conflict of interest.
